# Prognostic Value of VEGF in Patients Submitted to Percutaneous Coronary Intervention

**DOI:** 10.1155/2014/135357

**Published:** 2014-07-07

**Authors:** Catarina Ramos, Patrícia Napoleão, Mafalda Selas, Cláudia Freixo, Ana Maria Viegas Crespo, Miguel Mota Carmo, Rui Cruz Ferreira, Teresa Pinheiro

**Affiliations:** ^1^Instituto de Biociências e Bioengenharia (IBB), Instituto Superior Técnico, Universidade de Lisboa, Avenida Rovisco Pais 1, 1049-001 Lisboa, Portugal; ^2^Serviço de Cardiologia, Hospital de Santa Marta, Centro Hospitalar de Lisboa Central, 1069-024 Lisboa, Portugal; ^3^Carlota Saldanha Lab, Instituto de Medicina Molecular, Faculdade de Medicina da Universidade de Lisboa, 1649-028 Lisboa, Portugal; ^4^CESAM, Faculdade de Ciências, Universidade de Lisboa, 1749-016 Lisboa, Portugal; ^5^CEDOC, Faculdade de Ciências Médicas, Universidade Nova de Lisboa, 1169-056 Lisboa, Portugal

## Abstract

We examined the longitudinal changes of VEGF levels after percutaneous coronary intervention for predicting major adverse cardiac events (MACE) in coronary artery disease (CAD) patients. VEGF was measured in 94 CAD patients' serum before revascularization, 1-month and 1-year after. Independently of clinical presentation, patients had lower VEGF concentration than a cohort of healthy subjects (median, IQ: 15.9, 9.0–264 pg/mL versus 419, 212–758 pg/mL; *P* < 0.001) at baseline. VEGF increased to 1-month (median, IQ: 276, 167–498 pg/mL; *P* < 0.001) and remained steady to 1-year (median, IQ: 320, 173–497 pg/mL; *P* < 0.001) approaching control levels. Drug eluting stent apposition and previous medication intake produced a less steep VEGF evolution after intervention (*P* < 0.05). Baseline VEGF concentration <40.8 pg/mL conveyed increased risk for MACE in a 5-year follow-up. Results reflect a positive role of VEGF in recovery and support its importance in CAD prognosis.

## 1. Introduction

Vascular endothelial growth factor (VEGF) is an endothelial cell-specific mitogen. There are multiple direct and positive actions of VEGF in the endothelial function of adult resistance vessels. Some of the effects of VEGF stimulation on endothelial cells demonstrated* in vitro* and in animal models include cell motility and proliferation [1, 2], upregulation of endothelial nitric oxide synthase (NOS) [3], production of prostaglandins [4], modulation of apoptosis [5], permeability [6], protection mechanisms to manage increased shear stress [2, 7], and leukocyte adhesion [8], among others. VEGF signalling is required to stabilize the vasculature and to protect the endothelium by providing survival signals. The most important molecule regulating vascular development and angiogenesis in adults is the ligand VEGF-A (or VEGF) form, which exerts its actions via the VEGF receptor 1 (VEGFR-1) and VEGF receptor 2 (VEGFR-2).

It is currently accepted that VEGF plays an essential role in the normal vasculature and in the diseased vessels and that the mechanisms of action where VEGF intervenes are essentially different of those occurring in tumour angiogenesis [6, 9, 10].

VEGF has long been associated with coronary artery disease (CAD). The observation of VEGF release from platelets during clotting [11] together with the detection of increased VEGF mRNA expression in specimens of myocardium with evolving infarction but not in normal tissue [12] called the attention for the possible association of the levels of this protein with CAD phenotypes. However, the reported increases of VEGF expression in the myocardium following acute events were not unequivocally detected in the serum of patients; neither have the changes of VEGF levels in different clinical presentations reached a consensus [13–16].

The clinical correlations of systemic levels of VEGF in CAD and especially after revascularization intervention are largely unknown. Published data is scarce and only describes short-term changes of VEGF circulating levels after percutaneous coronary intervention (PCI) [17–19] or bypass surgery [13, 20] in small number of patients. Recently, emerging evidence suggests VEGF may have a protective net effect in the vasculature, giving rise to well-developed coronary collateral arteries in patients with CAD after revascularization intervention [21]. Therefore high VEGF levels would confer a better prognosis in CAD patients undergoing intravascular intervention as its actions may contribute to ameliorate the damaged endothelium and promote rapid recovery. The aim of this study was to investigate this hypothesis in CAD patients submitted to PCI by repeatedly measuring VEGF in a long-term basis, from baseline to 1-year and by examining the possible association of VEGF levels with future major adverse cardiac events (MACE) in a 5-year follow-up.

## 2. Methods

### 2.1. Study Population

This study included patients with coronary artery disease presented to the Cardiology Service of the Santa Marta Hospital (CHLC, Lisbon, Portugal) requiring invasive evaluation by PCI.

Ninety-four patients, men and women aged between 42 and 82, undergoing PCI for acute coronary syndromes (ACS) (*N* = 58)—ST-elevation and non-ST-elevation myocardial infarction and unstable angina—or for non-ACS (*N* = 36)—chronic stable angina—were prospectively eligible. ACS patients were admitted to the hospital in the first 6 hours after the onset of symptoms and enrolled in the study shortly after. Approximately 90% of the patients had stent placement (either drug eluting stent or bare metal stent types). All patients received standard care therapy including dual antiplatelet therapy after PCI.

Exclusion criteria were age above 85, myocardial infarction in the previous 5 years, peripheral artery disease or carotid artery disease, malignance or infectious diseases, chronic renal insufficiency, and concurrent inflammatory disorders.

A control group was constituted to help interpreting VEGF changes in patients from baseline to 1-year, as there are no reference values for the Portuguese population. The harmonization between controls and patients concerning age, gender, and risk factors was taken into account. Forty-four healthy volunteers were recruited at the preventive cardiology outpatient's clinic and enrolled in the study as controls. These individuals had no history of CAD and no signs or symptoms of coronary disease, based on ECG with negative stress test and normal endothelial function (ENDOPAT, peripheral arterial tonometry).

The CHLC Ethical Committee board approved the study protocol and all patients signed informed consent.

### 2.2. Longitudinal Study

Patients were evaluated 3 times over one-year: at admission and 1-month and 1-year after. At hospital admission, blood was drawn before PCI and any medication intake. At each evaluation point, core laboratory analysis of blood samples was performed for conventional tests and clinical chemistry, including N-terminal pro-brain natriuretic peptide (NT-proBNP) and C-reactive protein (CRP). All parameters were also measured in controls.

### 2.3. Follow-Up Evaluation and Study End Points

Patients were followed up annually for a period up to 5 years (24 ± 19 months) after the initial admission by clinic visits, telephone interviews, and analysis of hospital databases and clinical notes. The primary end points considered were death (with cardiac or procedure-related origin), ischemic events, hospital re-admission (for catheterization, PCI, or by-pass surgery), and CAD-related cardiovascular aggravation. The combination of primary end points rendered the secondary end point of major adverse cardiac events (MACE).

### 2.4. Quantification of Vascular Endothelial Growth Factor

Blood samples were processed within the hour after collection. Peripheral blood was centrifuged at 2500 rpm for 10 minutes. Serum was collected and stored at −80°C until analysis, for a period not exceeding 6 months. Samples were thawed only once. Serum concentrations of VEGF were measured by specific ELISA assays (designed to measure human VEGF-165 or VEGF-A) using the Quantikine Human VEGF kit (R&D Systems) according to the manufacturer's protocol. Each sample was measured in duplicate; intra-assay variation among the duplicates for all samples was <10%. The limit of detection of the assay was 9 pg/mL.

### 2.5. Statistical Analysis

Data were summarized and represented as median and interquartiles (IQ) 25% and 75% (Q25–Q75) for continuous variables and as proportions for categorical variables. The detection limit (DL) value of 9 pg/mL was assigned to samples with VEGF levels below DL. Categorical variables were analyzed using a 2 × 2 table and *χ* ^2^ test. Continuous variables were analysed using a Mann-Whitney test. Data consisted of repeated measurements on the same subject taken over time. To characterize the changes over time within-subjects, free of any between-subject variability and the variation in the time trends between-subjects, statistical linear mixed-effects models (LME) were used. The relationship between VEGF concentration in serum (response variable) through time (basic model) and the covariates observed or measured along with the response (as additional random effects), such as biochemical and clinical variables and medication, was evaluated. Examination of data indicated that it would be advantageous to take the square root transformation of VEGF, as the distribution for both groups of subjects becomes more symmetric and linearizes the relationship between VEGF and time. The model parameters and coefficients were estimated on the basis of the corresponding restricted maximum likelihood value (considered statistically significant for *P* < 0.05). Receiver operating characteristics (ROC) curve analyses over the dynamic range of the exposure variable were used to identify the threshold level granting highest predictive value for risk-stratifying patients with CAD. A Cox proportional-hazards regression analysis was performed for the primary and combined end points. Analyses were censored at 36 months. The calculations were performed using SPSS (version  21.0) and R (V.3.0.2).

## 3. Results

### 3.1. Demographics and Clinical Information at Baseline

Patients showed higher prevalence of arterial hypertension and diabetes than controls (see Table 1). The diagnostic markers of inflammation, CRP, cardiac dysfunction, and NT-proBNP were altered in patients. The circulating levels of both markers exceeded reference values and consequently differed from the levels measured in control individuals (Table 1). Over 78% of the enrolled patients took at least one prescription drug, whereas only 27% of the controls were on any medication.

### 3.2. Patient's Angiographic Information at Baseline

Angiographic and procedural findings were similar in ACS and non-ACS patients (Table 2). The presence of multivessel disease, the number of lesions in the left anterior descending artery, the length of lesions, and their morphological characteristics were identical in ACS and non-ACS patient's groups. ACS patients showed higher disease complexity with higher SYNTAX score values (range of 56), whereas non-ACS patients showed consistently lower scores (range value of 39; *P* < 0.001). Stents, DES or BMS type, were positioned in 73 patients.

### 3.3. VEGF Changes over 1-Year

The VEGF concentration in serum at baseline was decreased in CAD patients relative to controls (Table 1). No significant differences between ACS and non-ACS patients were observed (median, IQ: ACS = 16.6, 9.0–263 pg/mL; non-ACS = 18.2, 9.0–295 pg/mL; *P* > 0.05). Remarkably, 50% of ACS patients and 43% of non-ACS patients had VEGF concentration bellow the detection limit (9 pg/mL) at baseline. None of the controls had VEGF concentration below detection limit. No effect of any medication or risk factors on VEGF levels was observed, either considering all patients or ACS and non-ACS groups of patients. Also, there is no correlation between VEGF levels and cardiac or inflammatory markers, that is, NT-proBNP and CRP. Therefore, the VEGF concentrations in serum were significantly diminished in CAD patients, but their levels did not differ as a function of clinical presentation.

**Figure 1:**
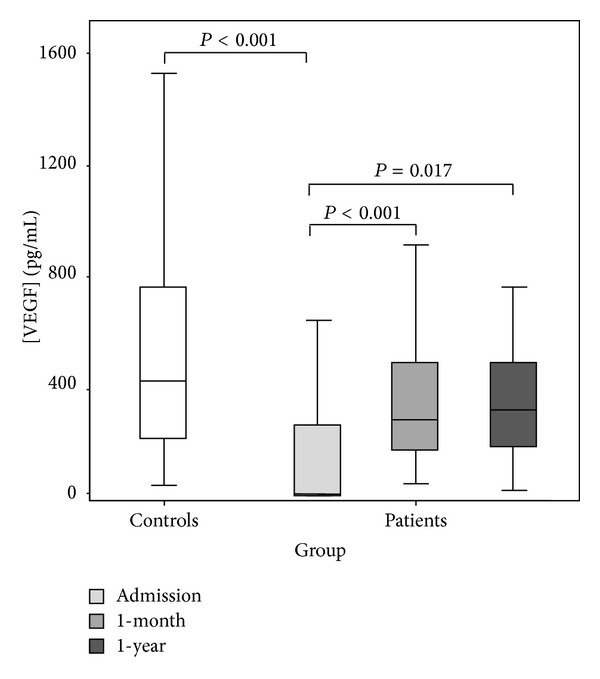
VEGF concentration in controls and in patients at admission, 1-month and 1-year after.

Patients were reevaluated 1-month and 1-year after intervention. The VEGF concentration showed a positive evolution through time in 84% of patients. The longitudinal changes of VEGF concentrations in the serum of patients significantly increased to 1-month and remained relatively steady to 1-year approaching the VEGF levels of controls (Figure 1). The interaction of type of stent positioned in the model of VEGF changes over time significantly influenced the average changes of VEGF from baseline to 1-year. The number of drug eluting stents (DES) positioned was correlated with a decrease in the average changes of VEGF to 1-year (*P* = 0.05). Counteracting this trend, patients carrying bare metal stents (BMS) showed a more steep average change in VEGF increases to 1-year (*P* = 0.04).

The model of VEGF changes over time was not significantly altered by the interaction of patient's clinical presentation (ACS and non-ACS), risk factors, angiographic characteristics, and specific biochemical indicators, such as CRP and NT-proBNP. Similar intercepts and slopes were produced indicating the lack of influence of the predictor variables on VEGF levels at the baseline and in the evolution.

### 3.4. Follow-Up Evaluation

**Figure 2:**
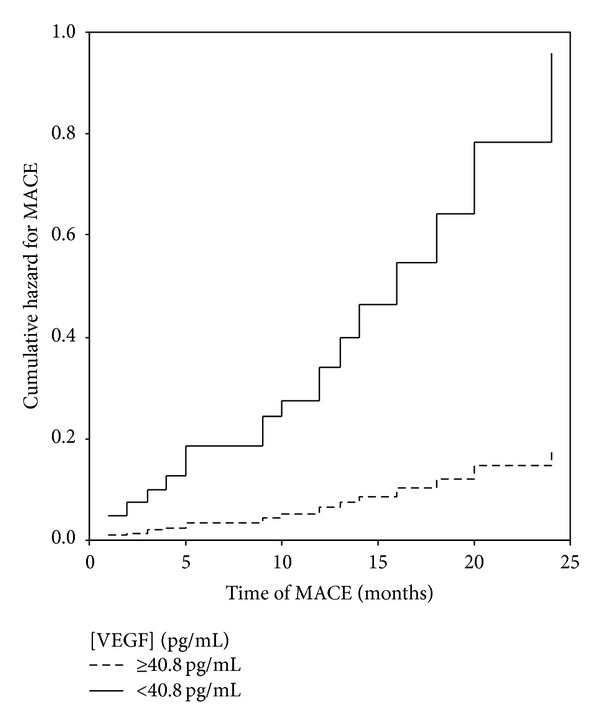
Cumulative hazard for MACE according to VEGF levels (<40.8 and ≥40.8 pg/mL).

The VEGF levels at admission were related to a 5-year follow-up analysis considering readmission (single end point) and MACE (composite end point).  Table 3 summarizes data for patients considering end points and the respective median time of event. Nineteen patients (28%) suffered MACE, with a median time of event of 10 months (IQ: 3–16). Lower VEGF concentrations at admission were significantly associated with patients with MACE compared to patients with higher VEGF: 9.0 versus 88.6 pg/mL; *P* = 0.028. Consistently with these results the ROC curve analysis indicated a cut-off level of 40.8 pg/mL for maximized predictive value for MACE (area under the curve of 0.664; 79% sensitivity; 56% specificity; *P* = 0.037). CAD group was dichotomized according to the calculated threshold level. Patients in the lower class of VEGF concentration showed increased risk for MACE, as represented in Figure 2 (Hazard Plot). The hazard ratio determined by a Cox regression was 4.4 (95% CI, 1.4 to 13.3; *P* = 0.009). Patients in the lower class of VEGF also showed increased risk for the primary end point of readmission (hazard ratio of 5.2; 95% CI, 1.1 to 24.1; *P* = 0.033).

## 4. Discussion

This study shows that the VEGF concentrations in the serum of CAD patients requiring PCI, either with acute or nonacute coronary syndromes, were lower than in healthy individuals without CAD or any history of CAD. VEGF levels increased over 1-year although 1-month after intervention the values approached those measured in controls. The VEGF concentration <40.8 pg/mL at admission was related to increased risk of MACE in a 5-year follow-up analysis.

A literature review of the clinical studies involving CAD patients showed that reported increases of VEGF in the circulation could be either beneficial or detrimental. VEGF changes in serum or plasma of patients with CAD relative to individuals without the disease varied from increased levels [15, 17] to decreased levels [16] or no changes [13, 22]. High VEGF levels were associated with patients who died of CAD [23] and with worst prognostic of ACS patients [14]. Opposite to these studies, decreased VEGF were associated with myocardial infarction history and CAD severity [24].

Our data are in line with the previous studies that have shown that VEGF increased through time after PCI. These studies have also evidenced the importance of the time gap of patient's evaluation on VEGF assessment. To our knowledge few studies have examined VEGF levels in CAD patients longitudinally after PCI [17–19]. None of previous studies reported on changes over 1-year period after stent apposition. However, consistent VEGF increases were reported on ACS and non-ACS patients after PCI as found in our study. In a small group of patients (*n* = 7) with acute myocardial infarction the VEGF increases were only significant 7 days after the intervention stabilizing to 180 days [17]. Banerjee et al. studied the variations of VEGF in a short period of 12 h after PCI and observed a faster VEGF increase in ACS patients when compared to non-ACS patients [18].

In our study, independently of clinical presentation, risk factors, and angiographic characteristics, there was a positive increasing trend in VEGF levels from baseline to 1-year, which was steeper to 1-month. However, previous medication intake influenced the rate of VEGF evolution to 1-month but not the values at the baseline. Also, drug eluting stent apposition produced a negative effect in the VEGF average changes after PCI evidencing a delay to reach control values to 1-year, which was not observed for bare metal stents. Decreases in VEGF levels were also reported on non-ACS patients treated either with statins [22] or with sirolimus eluting stent 1-month after apposition, but not in patients with bare metal stents [19]. By treating stenosis the ischemic trigger of angiogenesis is reduced [25] and this might be explained by the increase of VEGF levels [26]. This issue is relevant considering endothelial dysfunction after drug eluting stent apposition when compared with bare metal stent [27, 28].

Many observations suggest that endothelial cell dysfunction is crucially involved in the pathogenesis of atherosclerosis. Therefore, the consequences of low concentration levels of soluble VEGF in CAD patients must be discussed in the light of endothelial dysfunction. Moreover recent studies suggest that the mechanisms of action of VEGF in the normal vasculature and in the diseased vessels are different. At the ligand level, VEGF activities are controlled not only by its own level of expression, but also by the expression of various proteins that can bind and sequester it [29–32]. There are several evidences that VEGFR-1, existing either as a membrane bound form or as a soluble form [33], may act as a VEGF trap due to its poor kinase activity and high-affinity to VEGF, moderating the amount of free VEGF available to activate VEGFR-2 [34], the key receptor mediating most of cellular effects of VEGF in the endothelium [1, 2]. In fact, the overexpression of soluble VEGFR-1 was found to suppress phosphorylation of VEGFR-2 at specific sites* in vitro* and in placenta of preeclamptic women [34]. Also, Di Marco et al. demonstrated that the excess of VEGFR-1 in plasma of patients with chronic renal failure was associated with endothelial dysfunction and with cardiovascular risk [35]. These findings suggest that soluble VEGFR-1 would be more available in endothelial dysfunction conditions to sequester circulating VEGF. This hypothesis is in line with our findings of decreased VEGF levels in CAD patients at the baseline and of low VEGF concentrations (<40.8 pg/mL) in association with the increased risk of rehospitalization and combined adverse cardiac events in a 5-year follow-up. It should also be taken into account that the binding of VEGF to soluble VEGFR-1 in the circulation may mask the VEGF epitopes, hampering antigen detection by ELISA [36], which was the method used in this study and in the majority of published clinical studies. This would support the VEGF levels below detection limit in almost 50% of the ACS and non-ACS patients at the baseline, observed in our study. As normal endothelial function is restored the VEGF levels in patients approximated control values, which possibly reflect the physiological balance between free and bound VEGF in the circulation.

However, it would be crucial to study the interplay of VEGF soluble forms in the blood circulation of CAD patients. This will certainly improve the knowledge about the balance between VEGF ligands and receptors in health and disease and the understanding of their implications in the temporal control of signalling outputs.

## 5. Conclusion

In conclusion this study showed that VEGF levels in CAD patients progressively increase after revascularization during 1-year reaching the levels observed in controls. In addition, the increase in VEGF levels was associated with diminished occurrence of major adverse cardiac events for 5 years. Despite the limited number of patients evaluated in the study, results reflect a positive role of VEGF in endothelial function improvement after PCI.

## Figures and Tables

**Table 1:** Patients demographic, clinical, and biochemical characterization. Results are presented in median (Q25–Q75) unless otherwise specified.

Variable	Controls (*n* = 44)	Patients (*n* = 94)
Demographics		
Male sex (*n*, %)	28, 64	66, 70
Age (y)	58 (52–67)	62 (56–71)
Weight (kg)	72 (65–80)	76 (65–80)
Height (m)	1.6 (1.6–1.7)	1.6 (1.6–1.7)
Risk factors/comorbidities^a^		
Smoking (*n*, %)	4, 9	18, 20
Obesity (*n*, %)	25, 61	67, 74
Hypercholesterolemia (*n*, %)	22, 50	61, 66
Arterial hypertension (*n*, %)	17, 39	74, 80∗
Diabetes mellitus (*n*, %)	0	39, 42∗
Previous medication		
Aspirin (*n*, %)	4, 9	58, 64∗
ACE inhibitors (*n*, %)	9, 21	44, 49∗
Antiplatelets (*n*, %)	0, 0	48, 52∗
*β*-Blockers (*n*, %)	3, 7	41, 45∗
Statins (*n*, %)	10, 23	62, 68∗
Diagnostic markers		
CRP (mg/L)	1.6 (1–3.7)	4.3 (3.0–13.9)∗
NT-ProBNP (pg/mL)	33 (11–64)	148 (53–780)∗
VEGF (pg/mL)	419 (212–758)	15.9 (9.0–264)∗

^a^Diabetes: fasting plasma glucose concentration ≥7.0 mmol/L or 2 h plasma glucose ≥11.1 mmol/L or confirmed as clinically known and treated diabetes mellitus; hypertension: systolic blood pressure ≥140 mmHg or diastolic blood pressure ≥90 mmHg or use of antihypertensive therapy; dyslipidemia: total serum cholesterol ≥190 mg/dL or serum triglycerides ≥180 mg/dL or use of lipid-lowering medication; smoking: inhaled use of cigarettes, cigars, or pipes in any quantity, in the year previous to admission.

∗Significant differences to controls (*P* ≤ 0.01).

**Table 2:** Angiographic and procedural data expressed as within-group percentage of the total number of patients (%) unless otherwise specified.

	All patients	ACS	Non-ACS
Multivessel disease (*n*, %)	29, 31	19, 33	10, 29
Sintax Score^¥^	9.5 (3.5–17.5)	15.5 (9.5–22.8)	4.0 (0–8.8)∗
Intervened vessel (*n*, %)			
Left anterior descending artery	46, 51	29, 52	17, 50
Right coronary artery	28, 31	21, 38	7, 21
Left circumflex artery	11, 12	6, 11	5, 15
Left main artery	4, 4	0, 0	4, 12
Number of intervened lesions (*n*, %)			
1	52, 59	38, 68	14, 44
>1	20, 23	13, 23	7, 22
Lesion length (mm)^¥^	22 (16–30)	22 (16–35)	16 (13–26)
Long lesion (>15 mm) (*n*, %)	50, 77	36, 78	14, 74
Type B/C lesion (*n*, %)	55, 86	41, 89	14, 78
Eccentric lesion (*n*, %)	44, 75	29, 76	15, 71
Calcified lesion (*n*, %)	17, 21	13, 25	4, 13
Thrombotic lesion (*n*, %)	13, 16	12, 23	1, 3∗
Stent type (*n*, %)			
DES	50, 63	34, 65	16, 57
BMS	15, 19	11, 22	4, 14

^¥^Results expressed as median and interquartile (Q25–Q75).

∗Significant difference to ACS (*P* < 0.05).

**Table 3:** Patient's follow-up evaluation, according to study end-points. Time for event expressed as median (Q25–Q75).

Study end point	Positive cases (*n*, %)	Time for event (months)
Death	6, 9	3 (2–34)
Readmission	12, 18	13 (5–19)
Ischemic episode	6, 9	6 (1–15)
Cardiovascular aggravation	7, 10	∗
Composite end point MACE	19, 28	10 (3–16)

∗Based on annual clinical follow-up for 5-years.
